# Remedying the Metamemory Expectancy Illusion in Source Monitoring: Are there Effects on Restudy Choices and Source Memory?

**DOI:** 10.1007/s11409-022-09312-z

**Published:** 2022-08-10

**Authors:** Marie Luisa Schaper, Ute J. Bayen, Carolin V. Hey

**Affiliations:** Institute for Experimental Psychology, Heinrich-Heine-Universität Düsseldorf, 40225 Düsseldorf, Germany

**Keywords:** Metamemory Monitoring, Metamemory Control, Source Monitoring, Schemas, Delayed JOS

## Abstract

Metamemory monitoring, study behavior, and memory are presumably causally connected. When people misjudge their memory, their study behavior should be biased accordingly. Remedying *metamemory illusions* should debias study behavior and improve memory. One metamemory illusion concerns source memory, a critical aspect of episodic memory. People predict better source memory for items that originated from an expected source (e.g., toothbrush in a bathroom) rather than an unexpected source (e.g., shampoo in a kitchen), whereas actual source memory shows the opposite: an *inconsistency effect*. This *expectancy illusion* biases restudy choices: Participants restudy more unexpected than expected source–item pairs. The authors tested the causal relationships between metamemory and source memory with a delay and a source-retrieval attempt between study and metamemory judgment to remedy the expectancy illusion and debias restudy choices. Debiased restudy choices should enhance source memory for expected items, thereby reducing the inconsistency effect. Two groups studied expected and unexpected source–item pairs. They made metamemory judgments and restudy choices immediately at study or after delay, restudied the selected pairs, and completed a source-monitoring test. After immediate judgments, participants predicted better source memory for expected pairs and selected more unexpected pairs for restudy. After delayed judgments, participants predicted a null effect of expectancy on source memory and selected equal numbers of expected and unexpected pairs. Thus, the expectancy illusion was partially remedied and restudy choices were debiased. Nevertheless, source memory was only weakly affected. The results challenge the presumed causal relationships between metamemory monitoring, study behavior, and source memory.

## Remedying the Metamemory Expectancy Illusion in Source Monitoring: Are there Effects on Restudy Choices and Source Memory?

Metamemory includes the ability to assess and control one’s own learning and memory (Nelson & Narens, 1990). Interestingly, there are typical *metamemory illusions*, that is, people misjudge factors that affect their memory (Undorf, 2020). For example, people think that they will recall words written in large font better than words written in small font, although font size does not affect recall (Rhodes & Castel, 2008). Metamemory illusions offer insights into how people think about their memory, how they approach material to be studied, and why, sometimes, study behavior fails to enhance memory. Remedying metamemory illusions may potentially improve study behavior and, ultimately, improve memory. Based on presumed causal relationships between metamemory and memory, we investigated whether remedying a metamemory illusion about source memory improved study behavior (cf. Schaper & Bayen, 2021). Importantly, the experiment reported here is the first to test, in a source-monitoring paradigm, whether such improvement enhances memory.

Source monitoring refers to the cognitive processes involved when people attribute information to its source (Johnson et al., 1993). According to Johnson et al.’s source-monitoring framework, sources of information can be people, locations, modalities, font colors, study lists, etc. One important process in source monitoring is *source memory*, that is, remembering the source of information (e.g., who told you about an upcoming party, where you left your keys, or whether a studied word was shown at the top or bottom of the screen). Source memory is an important aspect of episodic memory and is vital for many everyday memory tasks. Source memory is affected by schema-based expectations (see review by Kuhlmann & Bayen, 2016). Schemas are knowledge frameworks about the world (Alba & Hasher, 1983). In daily life, almost every source elicits schema-based expectations about the type of information it will provide. For example, we expect a Republican politician to express conservative views. Critically for the study reported here, people misjudge the effect of such expectations on source memory. Source memory is better when information originates from a schematically unexpected source (e.g., shampoo located in a kitchen) than when it originates from a schematically expected source (e.g., toothbrush located in a bathroom; e.g., Küppers & Bayen, 2014). However, people are not aware of this *inconsistency effect* on source memory. To the contrary, they erroneously predict an *expectancy effect*, that is, that source memory benefits when information is expected for its source (Mieth et al., 2021; Schaper & Bayen, 2021; Schaper et al., 2019a, b, 2021; see also Konopka & Benjamin, 2009; Shi et al., 2012). This finding constitutes a *metamemory expectancy illusion* because memory and metamemory diverge. This expectancy illusion biases study behavior: People prefer to restudy unexpected source information, which they have already learned well at the expense of the less well-learned expected source information (Schaper & Bayen, 2021). In the experiment reported here, we investigated whether remedying this metamemory illusion debiased restudy choices and led people to restudy the source information they had not yet learned well. We then investigated whether such change in study behavior affected source memory.

### The Inconsistency Effect on Source Memory

The impact of schema-based expectations on source memory is typically investigated with the following paradigm. Participants study items that are presented with one of a set of sources. Items are either expected or unexpected for their source. For example, everyday object labels may appear in or with expected or unexpected scenes (e.g., “shower gel in the bathroom” vs. “frying pan in the bathroom”; Bayen et al., 2000; Küppers & Bayen, 2014; Mieth et al., 2021; Schaper & Bayen, 2021; Schaper et al., 2019a, b, 2021). In variants of this paradigm, a multitude of different item and source materials have been used (e.g., Arnold et al., 2013; Bayen & Kuhlmann, 2011; Bell et al., 2012, 2015; Dodson et al., 2008; Ehrenberg & Klauer, 2005; Hicks & Cockman, 2003; Kranz et al., 2019; Kroneisen & Bell, 2013; Kroneisen et al., 2015; Kuhlmann et al., 2012; Marsh et al., 2006; Mather et al., 1999; Mieth et al., 2016; Sherman & Bessenoff, 1999; Sherman et al., 1998; Spaniol & Bayen, 2002; Wulff & Kuhlmann, 2020). In a later source-monitoring test, the studied items are presented again, intermixed with distractors. Participants decide whether each item was previously presented with the expected source, the unexpected source, or not presented at all (cf. Johnson et al., 1993). This paradigm is referred to as the *schema-based source-monitoring paradigm*.

In this paradigm, source memory (corrected for guessing bias) shows an inconsistency effect. That is, source memory is typically better for items that originated from an unexpected source than for items that originated from an expected source (e.g., Bell et al., 2012, 2015; Ehrenberg & Klauer, 2005; Kranz et al., 2019; Kroneisen & Bell, 2013; Kroneisen et al., 2015; Küppers & Bayen, 2014; Mieth et al., 2016, 2021; Schaper & Bayen, 2021; Schaper et al., 2019a, b, 2021).^1^ This inconsistency effect has been explained by an attention-elaboration account (Brewer & Treyens, 1981; Erdfelder & Bredenkamp, 1998; Friedman, 1979; Küppers & Bayen, 2014; Loftus & Mackworth, 1978). According to this account, unexpected source–item pairs attract more attention during study than expected source–item pairs. Therefore, unexpected pairs are encoded more elaborately and remembered better, whereas expected pairs, which attract less attention during study, are encoded less elaborately, and the source is later remembered more poorly. The inconsistency effect on source memory stands in contrast to metamemory convictions.

### Metamemory Monitoring and Control of Schema-Based Source Memory

Metamemory includes monitoring and control. *Metamemory monitoring* refers to the assessment of one’s own memory. Metamemory monitoring is often measured via item-memory predictions called *Judgments of Learning* (JOLs; see review by Rhodes, 2016). In JOLs, participants predict the likelihood that they will later remember a presented item. In metamemory research on source memory, similar judgments are employed to measure source-memory predictions and are referred to as *Judgments of Source* (JOSs; see review by Kuhlmann & Bayen, 2016). In JOSs, participants predict the likelihood that they will later remember the source of a presented item.^2^*Metamemory control* refers to the measures a person takes to achieve a desired level of memory (e.g., Nelson & Narens, 1990). Metamemory control may be exerted, for example, through self-paced study times (e.g., Kornell & Finn, 2016) or restudy choices (e.g., Finley et al., 2010; Nelson et al., 1994). Metamemory monitoring, metamemory control, and memory are thought to be causally interconnected (Kornell & Finn, 2016). Important theoretical questions to be addressed by metamemory research are thus (1) whether and how people can accurately assess factors that benefit or impair memory, (2) whether they can account for such effects when controlling their study behavior, and (3) whether their controlled study behavior enhances memory.

A recent line of research on source monitoring has shown that people misjudge the effects of schemas on source memory to great extent: Whereas source memory shows an inconsistency effect, JOSs show an expectancy effect. This constitutes a metamemory expectancy illusion because memory and metamemory diverge. This illusion is robust and was shown with different source materials. It emerged when source–item expectancy was manipulated by pairing everyday object labels with scenes in which they were expected or unexpected (Schaper & Bayen, 2021; Schaper et al., 2019a, b, 2021) and when trustworthy- or untrustworthy-looking cheaters and cooperators served as sources (Mieth et al., 2021).^3^

Metamemory monitoring is generally theorized to guide metamemory control (e.g., Nelson & Narens, 1990; review by Kornell & Finn, 2016). Typically, people choose materials for restudy for which they predict poor memory, presumably with the aim to enhance memory for these materials (e.g., Kimball et al., 2012; Nelson et al., 1994).^4^ Accordingly, the expectancy illusion in source monitoring biased restudy choices (Schaper & Bayen, 2021). Participants’ prediction of an expectancy effect led them to choose more unexpected than expected source–item pairs for restudy. Metamemory control in turn is theorized to affect memory (Dunlosky & Metcalfe, 2009; Dunlosky et al., 2005, 2021; Nelson et al., 2004; Thiede, 1999; Thiede et al., 2003). In source monitoring, biased restudy choices coincided with the inconsistency effect on source memory (Schaper & Bayen, 2021): Participants who made biased restudy choices showed an inconsistency effect, whereas participants who restudied equal numbers of expected and unexpected source–item pairs (assigned by a computer program) did not. This pattern suggests a causal link between the metamemory expectancy illusion, biased restudy, and the inconsistency effect on source memory. Such a causal relationship between metamemory monitoring, metamemory control, and memory has been widely theorized (e.g., Dunlosky & Metcalfe, 2009; Dunlosky et al., 2005; Nelson et al., 2004; Thiede, 1999; Thiede et al., 2003).

Based on the presumed causal connection between metamemory monitoring, control, and source memory, we predicted that remedying the expectancy illusion would debias study choices and thereby enhance source memory for expected sources. Specifically, if people were able to predict the inconsistency effect on source memory (see Schaper et al., 2021), they should prioritize expected source–item pairs for restudy. In the study reported here, we added a crucial test of effects on memory: Restudying expected source–item pairs should in turn enhance source memory for these particular items, thereby attenuating the inconsistency effect on source memory.

### Remedying the Expectancy Illusion: Delaying Metamemory Judgments

To remedy the expectancy illusion in schema-based source monitoring, we need to understand its underlying mechanisms. Schaper et al. (2019b) showed that the expectancy illusion is based on two factors. First, participants base their JOSs on the in-the-moment experience of *processing fluency* at study (i.e., the subjective ease with which expected and unexpected source–item pairs are processed). Processing fluency is higher for expected pairs (Sherman et al., 1998) and people mistakenly equate this with better memory. Second, before the experiment, participants already hold the false *belief* that they will remember expected source–item pairs better, which also influences their JOSs. We can use the knowledge about these mechanisms to improve metamemory monitoring.

Metamemory monitoring can be improved via changes in belief and a shift to more diagnostic in-the-moment experiences by delaying metamemory judgments and eliciting retrieval of the to-be-remembered information before these judgments are made. Many studies have shown that delaying judgments positively affects judgment accuracy. That is, delayed judgments have a higher association with later memory than judgments rendered immediately at study (e.g., Bui et al., 2018; Dunlosky & Nelson, 1992, 1994, 1997; Kelemen, 2000; Kelemen & Weaver, 1997; Kimball & Metcalfe, 2003; Koriat & Bjork, 2006a, b; Koriat & Ma’ayan, 2005; Meeter & Nelson 2003; Nelson & Dunlosky, 1991; Nelson et al., 2004; Pyc et al., 2014; Tullis et al., 2013; Van Overschelde & Nelson, 2006; Weaver & Kelemen, 1997; for a meta-analysis, see Rhodes & Tauber 2011). Critically, delayed judgments only show this benefit if retrieval of the to-be-judged information is elicited before the judgment. For example, when people studied cue–target pairs in a paired-associate paradigm, delayed JOLs were more accurate than immediate JOLs if, after delay, the cue, but not the target, was presented (e.g., Dunlosky & Nelson, 1992; Kelemen, 2000; Tullis et al., 2013). Presumably, people engaged in target retrieval before rendering the delayed JOLs. Delayed judgments are more accurate than immediate judgments, presumably because, after target retrieval, people base their judgments on in-the-moment experiences that are predictive of later memory such as *retrieval fluency* (i.e., the subjective ease with which target information can be retrieved; Benjamin & Bjork, 1996).

Delaying metamemory judgments and eliciting retrieval can remedy metamemory illusions (e.g., Luna et al., 2018; Metcalfe & Finn, 2008; Yang et al., 2017). In Schaper et al. (2021), we showed that this also pertains to the expectancy illusion in source monitoring: Delaying JOSs and eliciting source retrieval remedied this illusion. In this prior study, we asked participants to render JOSs either immediately at study or after a delay. Preceding the delayed JOS, participants either saw the complete source–item pair (e.g., “oven in the kitchen”) or only the item (e.g., “oven”). If the complete pair was presented immediately preceding the (immediate or delayed) JOS, participants did not need to retrieve the source before the JOS because the source was presumably still available in short-term memory (cf. Nelson & Dunlosky, 1991). These participants predicted an illusory expectancy effect. If the item alone, but not the source, was presented preceding the delayed JOS, participants attempted to retrieve the source (either covertly, Experiment 1, or overtly, Experiment 2 and 3, Schaper et al., 2021). After the attempt to retrieve the source, they correctly predicted an inconsistency effect on source memory. Thus, the expectancy illusion was remedied. Schaper et al., (2021) showed that delaying JOSs and eliciting source retrieval changed the basis of participants’ judgments: Whereas immediate JOSs were based on processing fluency and an a priori general belief in an expectancy effect (Schaper et al., 2019b), delayed JOSs were based on the experience of retrieval fluency and an updated general belief. Retrieval can be assumed to be more fluent for items from unexpected than from expected sources (e.g., Schaper et al., 2021). This difference in retrieval fluency resulted in higher JOSs for unexpected than expected source–item pairs. Further, Schaper et al., (2021) showed that while rendering delayed JOSs, participants used their experience with source retrieval to change their initial belief in an expectancy effect toward the belief that expectations do not affect source memory. That is, participants’ false belief about the effect of schemas on source memory was partially remedied. Both retrieval fluency and participants’ beliefs about memory contributed to remedying the expectancy illusion in delayed JOSs. Therefore, in the study reported here, we delayed JOSs and elicited source retrieval to improve metamemory monitoring, metamemory control, and source memory. Hereafter, we refer to this condition as delayed JOS.

### The Current Study

This study is the first complete empirical test of all theorized causal relationships between metamemory monitoring, metamemory control, and source memory. Specifically, we tested whether remedying the expectancy illusion in schema-based source monitoring affected restudy choices and source memory. All participants studied expected and unexpected source–item pairs. We used two scenes (bathroom and kitchen) as sources and object labels as items. Participants were randomly assigned to two groups that differed in the timing of their JOSs. Participants in the *immediate-JOS* group made a JOS and a restudy choice immediately after studying each source–item pair. Participants in the *delayed-JOS* group completed an additional delayed-judgment phase during which they viewed the previously studied items again (but not the sources, cf. Schaper et al., 2021). These participants were asked to attribute the items to a source (bathroom or kitchen) to elicit source retrieval. They then made JOSs and restudy choices. Participants of both groups then restudied their chosen source–item pairs and completed a source-monitoring test.

We expected the immediate-JOS group to show the established metamemory expectancy illusion with higher JOSs for expected than unexpected source–item pairs (replicating Mieth et al., 2021; Schaper & Bayen, 2021; Schaper et al., 2019a, b, 2021). Due to the illusion, we expected the immediate-JOS group to choose more unexpected than expected source–item pairs for restudy (replicating Schaper & Bayen, 2021). By contrast, in the delayed-JOS group, we expected a remedy of the metamemory expectancy illusion (replicating Schaper et al., 2021), because participants had to attempt source retrieval before making a JOS and a restudy choice. This retrieval attempt should lead them to use retrieval fluency (higher for unexpected sources) or an updated belief about the effects of expectations on source memory to inform their JOSs. If the illusion is completely remedied, JOSs should be higher for unexpected than expected source–item pairs (i.e., an inconsistency effect). However, a partial remedy is also possible if the belief is only partially updated (i.e., it shows a null effect as reported by Schaper et al., 2021, Experiment 3). In this case, JOSs should not differ between unexpected and expected source–item pairs.

The first important innovation of our study was to test whether delayed JOSs informed restudy choices (Kimball et al., 2012; Nelson et al., 1994) and led to either selection of more expected source–item pairs for restudy (complete remedy) or selection of equal numbers of expected and unexpected pairs (partial remedy). The second, and even more critical, innovation of our study was to investigate whether or not metamemory control affected source memory. Source memory typically shows an inconsistency effect. This effect may be partially due to metamemory control that is biased by the expectancy illusion (Schaper & Bayen, 2021): Participants’ prioritization of unexpected source–item pairs for restudy may enhance source memory for unexpected pairs compared to expected pairs. Consequently, the immediate-JOS group should show a strong inconsistency effect on source memory. The delayed-JOS group, however, should show different effects on source memory. If the delayed-JOS group shows a different restudy pattern than the immediate-JOS group (i.e., more expected than unexpected pairs chosen for restudy or equal numbers chosen), their source memory may be affected accordingly. Restudy of a larger number of expected source–item pairs should increase source memory for items that were presented with their expected source. Therefore, the inconsistency effect on source memory should be attenuated or even eliminated in the delayed-JOS group.

## Methods

### Participants

The local ethics committee approved this research. We sought to detect an inconsistency effect on delayed JOSs, which was *d*_z_ = 0.35 in Experiment 3 by Schaper et al. (2021). A power analysis showed that 72 participants were needed to detect an effect of this size (one-sided, within subjects) with α = .05 and a power of .90. Therefore, we recruited 144 native German speakers (72 per group; 93 female, 51 male). Age ranged between 18 and 33 years (*M* = 23.15, *SE* = 0.27). Participants were assigned alternately to the two groups. We excluded and replaced one participant because they did not choose any source–item pairs for restudy.

### Design and Material

The experiment had a 2 × 2 mixed factorial design with JOS timing (immediate JOS, delayed JOS) as the between-subjects factor and source–item expectancy (expected, unexpected) as the within-subjects factor. Dependent variables were JOSs, restudy choices, and source memory. The materials and counterbalancing scheme were the same as in previous experiments (Schaper & Bayen, 2021; Schaper et al., 2019a, b, 2021). That is, items were 48 German object labels that were expected for a bathroom and unexpected for a kitchen, and 48 German object labels that were expected for a kitchen and unexpected for a bathroom (see norming study by Schaper et al., 2019a). There were three lists with 16 kitchen items and 16 bathroom items each that were equal in expectancy, number of syllables, and word frequency. At study, one list was assigned to the kitchen source and a second list was assigned to the bathroom source. Thus, participants studied 32 expected and 32 unexpected source–item pairs. The items from the third list served as distractors at test. The assignment of lists to conditions was counterbalanced within groups.

### Procedure

**Fig. 1:**
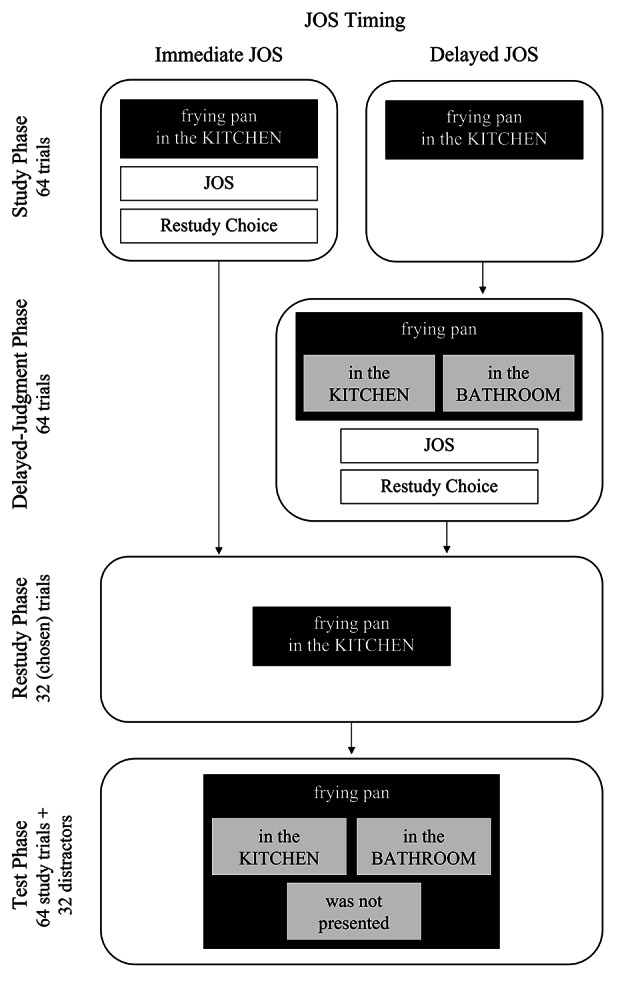
*Illustration of the Procedure* *Note*: The figure illustrates the four phases of the experiment with the exemplary expected source–item pair “frying pan in the KITCHEN” which was chosen to be restudied. Immediate JOS: Participants provided JOSs and restudy choices immediately at study. Delayed JOS: Participants provided JOSs and restudy choices after a delay.

We administered the experiment online to comply with physical-distancing regulations imposed due to the Covid-19 pandemic. Participants completed the experiment at home on their computers. They were proctored individually by an experimenter via a video call to ensure adherence to the study protocol. Figure 1 illustrates the procedure for both groups. It consisted of a study phase, a delayed judgment phase (in the delayed-JOS group only), a restudy phase, and a test phase.

#### Study Phase

Both groups were instructed to study source–item pairs that each consisted of an object label and a room. Participants were informed that they would later have to remember the object items and their sources. During the study phase, items (in standard German capitalization) and one of two sources (in all capitals, i.e. “in the KITCHEN” or “in the BATHROOM”) were presented in white letters on black background. Source–item pairs were presented one at a time in random order for 4 s each.

Immediately after the presentation of each source–item pair, the immediate-JOS group made a JOS followed by a restudy choice. All responses were self-paced. For the JOS, these participants judged the likelihood that they would later remember the item’s source (assuming perfect item memory). They were prompted with “Likelihood with which you will later remember in which ROOM (KITCHEN or BATHROOM) this object was located?” (room order counterbalanced) and made the JOS on a scale from 0% (“definitely will not remember”) to 100% (“definitely will remember”) using the computer keyboard. Upon response, the restudy choice screen appeared. Participants indicated whether or not they wanted to later restudy the pair by clicking on “yes” or “no” with the computer mouse. Prior to study, they were told that they could choose up to 32 out of 64 pairs, and would restudy 32 pairs regardless of how many they chose.^5^ During study, an on-screen tracker indicated how many pairs they had already studied and how many they could still choose for restudy. After choosing 32 pairs, they were informed that they had already chosen all pairs allowed rather than being shown the restudy choice screen. The delayed-JOS group made neither JOSs nor restudy choices during the study phase.

#### Delayed-Judgment Phase

Only the delayed-JOS group completed the delayed-judgment phase. After completion of the study phase, the participants of this group were informed of an upcoming two-answer forced-choice (2AFC) source-attribution task. They were told that they would view the same items from the study phase without their sources and should attribute each item to one of the two sources. They were further instructed to provide item-wise JOSs and restudy choices (with the same instructions the immediate-JOS group was given before their study phase). During the delayed-judgment phase, items were presented one at a time in a new random order for 4 s each, in white letters on black background. Below each item, two grey response boxes labeled “in the BATHROOM” and “in the KITCHEN” in black text were presented side by side (counterbalanced). To make their source attribution, participants clicked on the corresponding box with the computer mouse. There was no response feedback. They then provided their JOS and restudy choice.

#### Restudy Phase

Both groups completed the restudy phase. Thirty-two source–item pairs were again presented for 4 s each, in a new random order. Each item was shown with the same source with which it had been paired during the original study phase. Restudy choices were honored, that is, all participants restudied their chosen pairs. If they had chosen fewer than 32 pairs, the remainder was substituted with a random selection of 50% expected and 50% unexpected pairs that the participant had not chosen.

#### Test Phase

Finally, both groups completed a self-paced three-answer forced-choice (3AFC) source-monitoring test. In this test, the 64 studied items and 32 distractors were presented in random order. Participants indicated whether each item had been presented with the bathroom source, the kitchen source, or had not been presented. Below each item, two gray response boxes, labeled “in the BATHROOM” and “in the KITCHEN” in black text, were presented side by side (counterbalanced). A third response box was presented in the center beneath the other two and labeled “was not presented.” To answer, participants clicked on a box with the computer mouse. There was no response feedback. Finally, participants were debriefed and compensated. The total duration of the experiment was about 40 min.

## Results

We present two sets of analyses for metamemory monitoring and metamemory control. First, we tested the effect of expectancy of studied source–item pairs and its interaction with JOS timing (immediate JOS, delayed JOS) on JOSs and restudy choices. Second, we present results from the 2AFC source-attribution task which the delayed-JOS group completed in the delayed-judgment phase. Finally, we present the results on source memory. The data are accessible from the Open Science Framework at https://osf.io/h6ma8.

### Metamemory Monitoring and Metamemory Control

#### Effects of Expectancy and JOS timing on JOSs and restudy choices

**Fig. 2:**
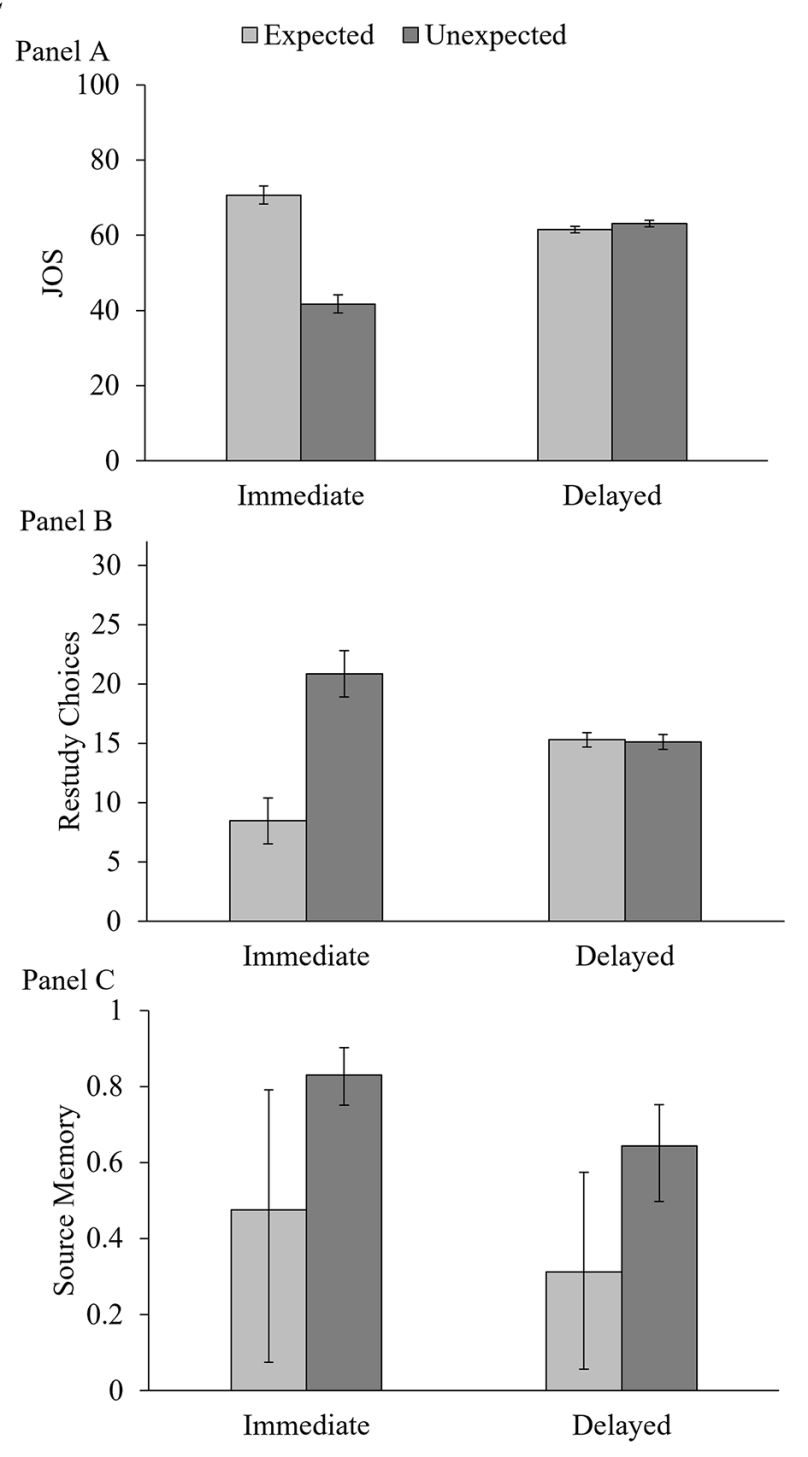
*Effects of Expectancy and JOS Timing on JOSs, Restudy Choices, and Source Memory* *Note*: The effects of expectancy and JOS timing on JOSs are shown in Panel A, on restudy choices in Panel B, and on source memory in Panel C. Immediate JOS: Participants provided JOSs and restudy choices immediately at study. Delayed JOS: Participants provided JOSs and restudy choices after a delay. For JOSs and restudy choices, error bars represent 95% within-subjects confidence intervals (Loftus & Masson, 1994). In Panel C, source memory is the probability that a participant remembers the expected/unexpected source of an item (as measured by probability parameter *d*). Here, error bars represent 95% Bayesian credibility intervals.

**JOSs.** Figure 2 (Panel A) shows descriptive statistics for JOSs. We performed a 2 × 2 mixed ANOVA with the within-subjects factor expectancy (expected vs. unexpected) and the between-subjects factor JOS timing (immediate JOS, delayed JOS). There were main effects of expectancy, *F*(1, 142) = 118.04, *p* < .001, η_p_^2^ = .45, and JOS timing, *F*(1, 142) = 7.75, *p* = .006, η_p_^2^ = .05. Critically, the results were strongly qualified by the predicted two-way interaction between expectancy and JOS timing, *F*(1, 142) = 147.40, *p* < .001, η_p_^2^ = .51. Due to this interaction, the main effects cannot be globally interpreted. We performed follow-up paired-samples *t* tests to examine the interaction. In the immediate-JOS group, JOSs were higher for expected than unexpected pairs, *t*(71) = 12.23, *p* < .001, *d*_z_ = 1.44. This group thus replicated the established expectancy effect on immediate JOSs (Mieth et al., 2021; Schaper & Bayen, 2021; Schaper et al., 2019a, b, 2021). In the delayed-JOS group, JOSs were numerically higher for expected than unexpected pairs (i.e., they showed a numerical inconsistency effect). However, this inconsistency effect narrowly missed the conventional level of significance, *t*(71) = 1.88, *p* = .064, *d*_z_ = 0.22. Overall, delaying JOSs only partially remedied the metamemory expectancy illusion (Schaper et al., 2021) as the delayed-JOS group did not quite predict an inconsistency effect on source memory.

**Restudy Choices.** The immediate-JOS group selected *M* = 29.31 source–item pairs for restudy (*SE* = 0.62, *median* = 32.00), and the delayed-JOS group selected *M* = 30.40 items for restudy (*SE* = 0.34, *median* = 32.00). Figure 2 (Panel B) shows descriptive statistics by JOS timing and expectancy. Mirroring the analyses for JOSs, we performed a 2 × 2 mixed ANOVA with the factors expectancy and JOS timing on restudy choices. There was no main effect of JOS timing, *F*(1, 142) = 2.43, *p* = .121, η_p_^2^ = .02, indicating that, overall, there was no group difference in the number of pairs chosen for restudy. There was a main effect of expectancy, *F*(1, 142) = 35.29, *p* < .001, η_p_^2^ = .20, which was strongly qualified by a two-way interaction, *F*(1, 142) = 37.41, *p* < .001, η_p_^2^ = .21. We used follow-up paired-samples *t* tests to examine the interaction. The immediate-JOS group selected more unexpected than expected source–item pairs for restudy, *t*(71) = 6.33, *p* < .001, *d*_z_ = 0.75. This aligns with the prediction of an expectancy effect in their JOSs and replicates the results by Schaper and Bayen (2021). The delayed-JOS group selected equal numbers of expected and unexpected pairs for restudy, *t*(71) = 0.29, *p* = .773, *d*_z_ = 0.03. This aligns with the prediction of a null effect of expectancy on source memory in their JOSs.

**Relationship between JOSs and Restudy Choices.** We assessed the relationship between JOSs and restudy choices with Kruskal-Goodman gamma correlations (Nelson, 1984; coding restudy choices as 0 for “no” and as 1 for “yes”). Participants chose those source–item pairs for which they predicted worse source memory, both in the immediate-JOS group^6^, mean γ = −.58, *t*(70) = 8.71, *p* < .001, *d* = 1.03, and in the delayed-JOS group, mean γ = −.35, *t*(71) = 4.12, *p* < .001, *d* = 0.49. Interestingly, an independent-samples *t* test of the gamma correlations showed that the correlation between JOSs and restudy choices was stronger in the immediate-JOS group than in the delayed-JOS group, *t*(141) = 2.03, *p* = .045, *d* = 0.34. Thus, in the immediate-JOS group, JOSs were biased by an illusory expectancy effect and participants relied heavily on these inaccurate JOSs to guide their restudy choices. In the delayed-JOS group, JOSs were not biased as strongly (i.e., were more accurate) and participants relied on them to a lesser degree to guide their restudy choices.

#### Effects of Source Attribution and Attribution Accuracy on Delayed JOSs and Restudy Choices

The delayed-JOS group was required to make 2AFC source attributions before JOSs and restudy choices. We expected the accuracy of these source attributions to affect JOSs (Schaper et al., 2021) and, consequently, restudy choices. First, we expected participants to provide higher JOSs in trials with correct source attributions and to make more restudy choices in trials with incorrect source attributions. Second, we sought to further investigate the remediation of the expectancy illusion. Schaper et al.’s (2021) participants predicted an inconsistency effect on source memory, especially in trials with correct source attributions. We, therefore, tested for an inconsistency effect in trials with correct and incorrect source attributions. In the following sections, we first describe the 2AFC source attributions. Then, we present the effects of attribution accuracy (correct, incorrect) and source attribution (to the expected or unexpected source) in the delayed-judgment phase on JOSs and restudy choices.

**2AFC Source Attributions in the Delayed-Judgment Phase.** Table 1 shows the absolute frequencies (aggregated across participants and items) of correct and incorrect source attributions for items from expected and unexpected trials in the delayed-judgment phase. As expected, a paired-samples *t* test showed more correct attributions (68%) than incorrect attributions (32%), *t*(71) = 13.07, *p* < .001, *d*_z_ = 1.54. Further, a paired-samples *t* test showed more correct attributions of items to their expected source (75%) than to their unexpected source (61%), *t*(71) = 4.37, *p* < .001, *d*_z_ = 0.52. This is a common result that can be explained by a well-documented schema-consistent guessing bias (see review by Kuhlmann & Bayen 2016, and the results from the 3AFC source-monitoring task reported in Table 2).

**Table 1:** Response Frequencies

JOS timing	Task	Trial Type	Response		
			“Expected”	“Unexpected”	“New”
Immediate JOS	3AFC	Expected source	**1632**	248	424
		Unexpected source	398	**1483**	423
		New	160	67	**2077**
Delayed JOS	2AFC	Expected source	**1732**	572	
		Unexpected source	898	**1406**	
	3AFC	Expected source	**1556**	429	319
		Unexpected source	498	**1528**	278
		New	124	69	**2111**

*Note.* Response frequencies are aggregated across items and participants. The correct responses are in bold print. For each trial type in each group, the total number of observations is 2,304 (= 32 items × 72 participants). Immediate JOS: Participants provided JOSs and restudy choices immediately at study. Delayed JOS: Participants provided JOSs and restudy choices after a delay. 2AFC: Two-answer forced-choice (expected or unexpected) source attributions in the delayed-judgment phase of the delayed-JOS group. 3AFC: Three-answer forced-choice (expected, unexpected, new) source-monitoring task. For the 2AFC task, the “New” cells are empty because there were no new items in this task.

**Table 2:** Parameter Estimates of the Multinomial Model of Source Monitoring

Parameter	JOS timing	
	Immediate JOS	Delayed JOS
*D*_E_	.71 [.66, .76]	.78 [.74, .82]
*D*_U_ = *D*_N_	.73 [.69, .77]	.83 [.79, .86]
*d*_E_	.48 [.08, .79]	.31 [.06, .58]
*d*_U_	.83 [.75, .90]	.64 [.50, .75]
*b*	.33 [.25, .40]	.39 [.30, .48]
*g*	.75 [.66, .82]	.61 [.50, .70]

*Note.* Parameter estimates are probabilities in the interval [0, 1]. *D*_E_/*D*_U_ = the probability that an item from the expected/unexpected source is recognized as old. *D*_N_ = the probability that a participant knows that a new item is new. *d*_E_/*d*_U_ = the probability that a participant remembers the expected/unexpected source of an item. *b* = the probability that a participant guesses that an item is old. *g* = the probability that a participant guesses that an item was presented with the source for which it was expected. Immediate JOS: Participants provided JOSs and restudy choices immediately at study. Delayed JOS: Participants provided JOSs and restudy choices after a delay. 95% Bayesian credibility intervals are given in brackets.

**Fig. 3:**
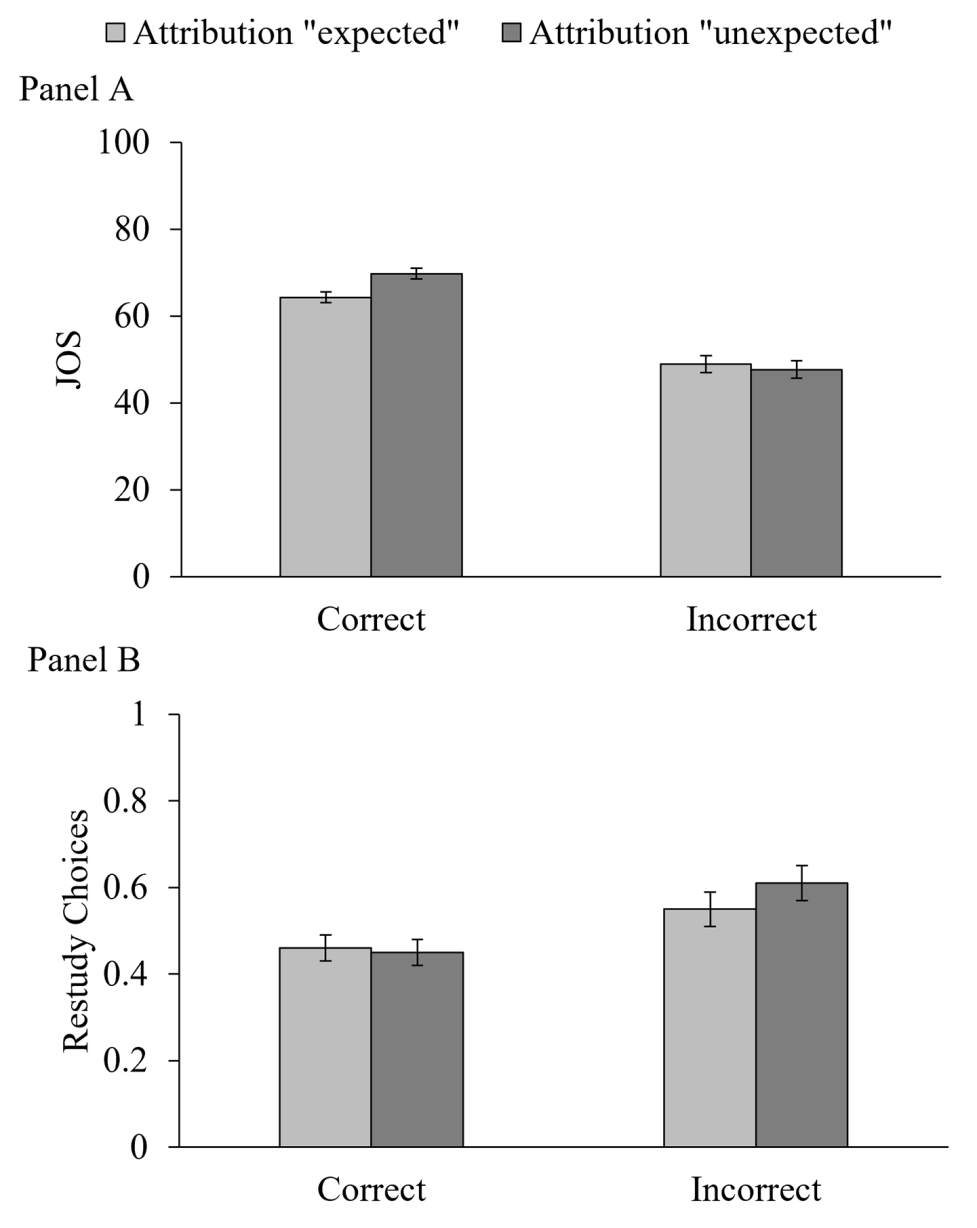
*Effects of Source Attribution and Attribution Accuracy on Delayed JOSs and Restudy Choices* *Note*: The effects of source attribution and attribution accuracy in the delayed-JOS group on JOSs are given in Panel A and on restudy choices in Panel B. The relative frequencies of restudy choices are shown. Error bars represent 95% within-subjects confidence intervals (Loftus & Masson, 1994).

**JOSs.** Figure 3 (Panel A) shows descriptive statistics for JOSs. We performed a 2 × 2 within-subjects ANOVA with the factors attribution accuracy (correct, incorrect) and source attribution (to the expected or unexpected source).^7^ As expected, there was a main effect of attribution accuracy, *F*(1, 70) = 147.49, *p* < .001, η_p_^2^ = .68, such that participants provided higher JOSs when their source attribution was correct than when it was incorrect. Thus, participants had some insight into the accuracy of their source monitoring during the delayed-judgment phase. There was no main effect of source attribution, *F*(1, 70) = 2.57, *p* = .114, η_p_^2^ = .04. There was a two-way interaction, *F*(1, 70) = 9.89, *p* = .002, η_p_^2^ = .12, which we followed up with paired-samples *t* tests. In trials with correct source attributions, JOSs were higher for attributions to the unexpected than for attributions to the expected source, *t*(71) = 4.37, *p* < .001, *d*_z_ = 0.52. Thus, participants predicted an inconsistency effect in delayed JOSs if they had correctly attributed the item to its source before JOS (replicating Schaper et al., 2021). By contrast, in trials with incorrect source attributions, JOSs did not differ after attributions to the expected source and attributions to the unexpected source, *t*(70) = 0.64, *p* = .526, *d*_z_ = 0.08.

**Restudy Choices.** Figure 3 (Panel B) shows descriptive statistics for restudy choices. To account for the different numbers of observations in the four cells, we calculated the relative frequency of restudy choices in each cell. For example, if a participant correctly attributed 24 items to the expected source, and chose 12 of these items for restudy, the relative frequency of restudy choices for trials with correct source attributions would be .50 (see Footnote 6). Mirroring the analysis of JOSs, we performed a 2 × 2 within-subjects ANOVA with the factors attribution accuracy (correct, incorrect) and source attribution (to the expected or unexpected source). Mirroring the results for JOSs, there was a main effect of attribution accuracy, *F*(1, 70) = 11.60, *p* = .001, η_p_^2^ = .14, indicating that participants chose those source–item pairs more often for restudy for which they made incorrect source attributions. This is a reasonable strategy because these pairs were not yet well-learned. There was no main effect of expectancy, *F*(1, 70) = 0.90, *p* = .347, η_p_^2^ = .01 and no interaction *F*(1, 70) = 2.23, *p* = .140, η_p_^2^ = .03. Thus, restudy choices did not differ between attributions to the expected versus the unexpected source, although JOSs were higher for correct attributions to the unexpected source. The gamma correlations between JOSs and restudy choices were not affected by attribution accuracy or source attribution (all *p*s > .053).

### Source Memory

#### Measuring Source Memory

**Fig. 4:**
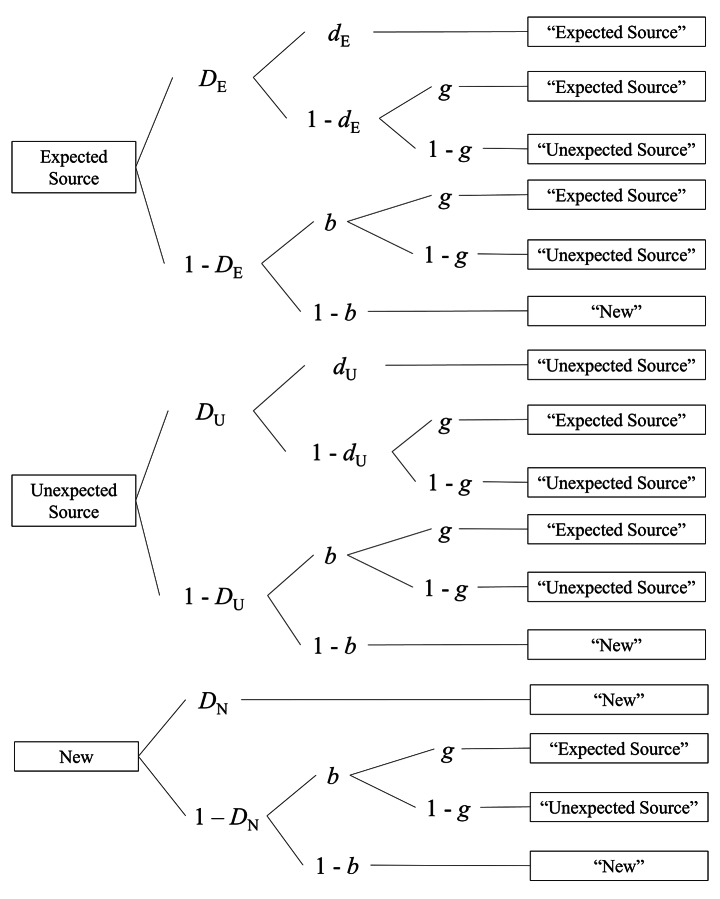
*Two-High-Threshold Multinomial Model of Source Monitoring* *Note*: *D*_E_/*D*_U_ = the probability that an item from an expected/unexpected source is recognized as old. *D*_N_ = the probability that a participant knows that a new item is new. *d*_E_/*d*_U_ = the probability that a participant remembers the expected/unexpected source of an item. *b* = the probability that a participant guesses that an item is old. *g* = the probability that a participant guesses that an item was presented with the source for which it was expected. This version of the model assumes that source guessing is equal for recognized and unrecognized items. Adapted from “Source Discrimination, Item Detection, and Multinomial Models of Source Monitoring,” by Bayen et al., 1996, Journal of Experimental Psychology: Learning, Memory, and Cognition, 22, p. 202, Fig. 3. Copyright 1996 by the American Psychological Association.

To answer our research question, we compared and contrasted metamemory monitoring and control with source memory in the final 3AFC source-monitoring task. Critically to this end, we needed a pure measure of source memory. Multiple cognitive processes play a role in 3AFC source-monitoring tasks: Performance in such tasks is influenced by item memory, source memory, and guessing (Batchelder & Riefer, 1990; Bayen et al., 1996; Bröder & Meiser, 2007; Murnane & Bayen, 1996, 1998). Source–item expectancy affects memory and guessing differentially (see review by Kuhlmann & Bayen, 2016). Therefore, memory must be measured independent of guessing. Traditional behavioral measures of source-attribution performance (e.g., correct source attributions conditionalized on item hits; Bröder & Meiser, 2007; Murnane & Bayen, 1996) confound source memory and source guessing. The multinomial processing-tree model of source monitoring (Bayen et al., 1996) solves this problem because it allows the separate measurement of the cognitive processes at play in 3AFC source-monitoring tasks. Figure 4 shows the model. The first tree refers to items presented with the expected source, the second tree refers to items presented with the unexpected source, and the third tree refers to new items.

Participants recognize an item that was presented with the expected source (first tree) with probability *D*_E_ (item memory). In this case, they remember that the item was presented with the expected source with probability *d*_E_ (source memory), resulting in a correct response. With probability 1 – *d*_E_, participants do not remember the source and must guess a source. With probability *g*, they guess that the item was presented with the expected source (source guessing). With probability 1 – *g*, they guess that the item was presented with the unexpected source. With probability 1 – *D*_E_, participants do not recognize the item, and guess, with probability *b*, that it is old. They further guess that the item was presented with the expected source (with probability *g*) or with the unexpected source (with probability 1 – *g*). With probability 1 – *b*, participants guess that an item is new. The other model trees follow the same logic but contain different item-memory and source-memory parameters for items presented with the unexpected source (*D*_U_ and *d*_U_, respectively) and new items (*D*_N_). To obtain an identifiable model, an equality restriction must be imposed on parameter *D*_N_ (Bayen et al., 1996). We set *D*_N_ = *D*_U_ (cf. Schaper et al., 2019a). Note that if instead, we set *D*_N_ = *D*_E_, the conclusions regarding source memory are the same.

Table 1 shows the response frequencies from the source-monitoring task, aggregated across participants within group. We used the Bayesian hierarchical latent-trait approach (Klauer, 2010) to estimate model parameters for item memory (*D*), source memory (*d*), old/new guessing (*b*), and source guessing (*g*) from individual response frequencies. As Schaper et al. (2021) explained, “the latent trait approach draws participant parameters from a multivariate normal distribution of probit transformed parameters. Via Bayesian modeling, the a priori distribution is updated to a posterior distribution for each parameter given the data (Bayes’ theorem). The Markov chain Monte Carlo (MCMC) algorithm draws samples from the posterior parameter distribution. Thus, individual parameter estimates and corresponding parameter distributions are obtained. The parameter distribution is characterized by the Bayesian Credibility Interval which indicates the interval in which the true parameter can be found with 95% confidence.” (p. 25).

We performed Bayesian-hierarchical parameter estimation with the R package TreeBUGS (for a tutorial, see Heck et al., 2018). TreeBUGS uses the MCMC algorithm implemented in JAGS (Plummer, 2003). We used 500,000 samples with a burn-in period of 250,000 samples. We retained every tenth sample. We assessed parameter convergence with the Gelman-Rubin statistic $\widehat{R}$ (Gelman & Rubin, 1992). All parameters showed good convergence (all $\widehat{R}$ < 1.01). We assessed model fit with the *T*_1_ and *T*_2_ test statistics (Klauer, 2010). Non-significant test results indicate a good model fit. Model fit was good for both the immediate-JOS group (*T*_1_: *p* = .513, *T*_2_: *p* = .455) and the delayed-JOS group (*T*_1_: *p* = .479, *T*_2_: *p* = .400). In the next section, we report the results for the group-wise source-memory parameters. The full set of model parameters is presented in Table 2. In the Appendix, we report the results on source guessing.

#### Effects of Expectancy and JOS Timing on Source Memory

To answer our research question, we focused on source memory (model parameters *d*_E_ for expected source and *d*_U_ for unexpected source). We sought to determine whether different restudy choices in the two groups affected the inconsistency effect on source memory. As reported above, participants in the immediate-JOS group predicted an expectancy effect on source memory and, accordingly, selected more unexpected source–item pairs for restudy. Therefore, we expected a strong inconsistency effect on source memory in this group (cf. Schaper & Bayen, 2021). By contrast, participants in the delayed-JOS group predicted a null effect and selected equal numbers of expected and unexpected source–item pairs for restudy. Based on these restudy choices, we expected better source memory for expected pairs in the delayed-JOS group than in the immediate-JOS group. This should attenuate or eliminate the inconsistency effect on source memory in the delayed-JOS group.

Figure 2 (Panel C) shows the parameter estimates for source memory. We first tested whether source memory for expected items (parameter *d*_E_) and unexpected items (parameter *d*_U_) differed between the two groups. To this end, we calculated the parameter differences Δ*d*_E_ = *d*_E_immediate_ – *d*_E_delayed_ and Δ*d*_U_ = *d*_U_immediate_ – *d*_U_delayed_. A parameter difference is considered significant if the 95% Bayesian Credibility Interval (given in brackets) does not contain zero. Somewhat surprisingly, despite different restudy behavior, source memory for expected items did not differ significantly between groups, Δ*d*_E_ = .16 [–.32, .59], and was descriptively better in the immediate-JOS group. Source memory for unexpected items was better in the immediate-JOS group, Δ*d*_U_ = .19 [.05, .35]. This latter finding is consistent with the differential restudy behavior between groups: Participants in the immediate-JOS group restudied more unexpected pairs than participants in the delayed-JOS group which may explain their better source memory for unexpected items.

We then tested for inconsistency effects on source memory (i.e., better source memory for items originating from an unexpected source) in the two groups by calculating the parameter difference Δ*d* = *d*_U_ – *d*_E_. As expected, the immediate-JOS group showed an inconsistency effect, Δ*d* = .36 [.01, .77]. Surprisingly, the delayed-JOS group also showed an inconsistency effect, Δ*d* = .33 [< .01, .64]. The strength of the effect did not differ between groups, Δ_inconsistency effect_ = .02 [–.46, .56]. Thus, despite the restudy differences between groups, the inconsistency effect on source memory appeared in both groups. Remedying the metamemory expectancy illusion and debiasing corresponding restudy choices did not eliminate or attenuate the inconsistency effect on source memory.

## Discussion

According to theories of metamemory, it is often assumed that metamemory monitoring, control, and memory are causally connected (e.g., Nelson & Narens, 1990). In metamemory monitoring, people severely misjudge some factors that affect memory. Such metamemory illusions may result in biased, ineffective study behavior. Due to the presumed causal relationships between metamemory monitoring, control, and memory, remedying metamemory illusions thus seems promising to debias study behavior and, ultimately, improve memory. In this research, we sought to remedy the expectancy illusion in schema-based source monitoring by implementing a delay between study and JOSs and by eliciting source retrieval before the JOSs (replicating Schaper et al., 2021). Restudy choices after a delay were less biased by schema-based expectations than restudy choices immediately at study. However, although the delayed-JOS group restudied more expected source–item pairs than the immediate-JOS group, source memory for expected sources did not benefit from this restudy. Independent of restudy choices, both groups showed an inconsistency effect on source memory. Thus, remedying the expectancy illusion in source monitoring debiased restudy choices, but differential restudy choices only weakly affected source memory. These results challenge some of the presumed causal relationships among metamemory monitoring, metamemory control, and source memory. In the following, we first discuss the remediation of the expectancy illusion in metamemory monitoring and control, and then the lack of effects on source memory.

### Improving Metamemory Monitoring and Control in Source Monitoring

Delaying metamemory judgments and eliciting retrieval of the to-be-learned information improves metamemory accuracy in general (see meta-analysis by Rhodes & Tauber, 2011) and has been shown to remedy the expectancy illusion in source monitoring specifically (Schaper et al., 2021). Therefore, we used this procedure to remedy the expectancy illusion. The immediate-JOS group showed the established expectancy effect on JOSs (Mieth et al., 2021; Schaper & Bayen, 2021; Schaper et al., 2019a, b, 2021), which did not concur with the inconsistency effect on their source memory. This group thus replicated the expectancy illusion in source monitoring.

Remedying the expectancy illusion in the delayed-JOS group was partially successful. In contrast to the immediate-JOS group, the delayed-JOS group showed a null effect on delayed JOSs and a numerical inconsistency effect. This prediction of a null effect aligned more with the inconsistency effect on source memory than the immediate-JOS group’s prediction of an expectancy effect. However, the expectancy illusion was not fully remedied as the delayed-JOS group did not accurately predict the inconsistency effect on source memory. In Schaper et al.’s (2021) experiments, delaying JOSs fully remedied the expectancy illusion. However, the inconsistency effect on delayed JOSs reported there was comparably small, suggesting that delaying JOSs did not fully reverse the expectancy/inconsistency prediction. Furthermore, we split delayed JOSs by correct versus incorrect attribution in the preceding 2AFC source-attribution task. Importantly, only when the attribution before the JOS was correct, participants accurately predicted an inconsistency effect on source memory. Correspondingly, in the prior study (Schaper et al., 2021), we also found a stronger inconsistency effect on delayed JOSs for correctly attributed items. Thus, overall, delayed JOSs were markedly less biased by schema-based expectations than immediate JOSs, but the delay did not fully remedy the expectancy illusion.

The improvement in metamemory monitoring by means of delayed JOSs was likely due to a change in the metamemory cues used in JOS formation (cf. Koriat, 1997). As shown previously, immediate JOSs are based on processing fluency (which is higher for expected source–item pairs) and the incorrect general belief that source–item expectancy benefits source memory (Schaper et al., 2019b). Delayed JOSs, by contrast, are based on retrieval fluency (which is higher for unexpected pairs) and an updated belief about effects of source–item expectancy (Schaper et al., 2021). The delayed-JOS group of the current experiment presumably experienced their source memory by attempting source retrieval before the delayed JOSs. They may have noticed that source memory was better for unexpected than expected source–item pairs and consequently updated their belief about the impact of schema-based expectations on source memory. In the experiments we reported in Schaper et al. (2021), this belief update was partial: Participants corrected their belief from illusory expectancy toward a null effect but did not fully update it to an inconsistency effect. Reliance on such a partially updated belief may explain why the delay in the current experiment merely led to an overall null effect of schema-based expectations on JOSs.

As predicted, the between-group differences in metamemory monitoring translated into differences in metamemory control. The immediate-JOS group chose to restudy more unexpected than expected source–item pairs, presumably because they predicted an expectancy effect on source memory. Thus, as in previous research, participants chose materials for restudy for which they predicted poorer memory (Kimball et al., 2012; Nelson et al., 1994; Schaper & Bayen, 2021) likely intending to enhance their memory for materials they had not yet learned well. The delayed-JOS group, by contrast, chose to restudy equal numbers of expected and unexpected source–item pairs. These restudy choices aligned with the null effect of expectancy on delayed JOSs. The delayed-JOS group presumably predicted that source memory would be unaffected by schema-based expectations and, accordingly, chose to restudy equal numbers of expected and unexpected pairs. Interestingly, when we split restudy choices in the delayed-JOS group by correct versus incorrect attribution in the preceding 2AFC source-attribution task, participants more frequently chose items for restudy when their source attribution had been incorrect. This strategy aligned with their JOSs, which were lower for incorrectly attributed items. The results show that the delayed-JOS group gained some insight into their memory. They noticed to some extent when they had made an incorrect attribution and chose to restudy the corresponding source–item pair, presumably to enhance source memory on the subsequent test.

However, the delayed-JOS group did not fully capitalize on their improved metamemory monitoring to inform their restudy choices. First, restudy choices were unaffected by whether participants attributed an item to the expected or unexpected source. This contrasts with JOSs: In delayed JOSs, participants predicted an inconsistency effect when they had made a correct source attribution. However, this inconsistency prediction did not translate into restudy choices. Regardless of attribution accuracy, whether participants attributed an item to the expected or unexpected source did not affect restudy choice.

Second, in both groups, there were negative gamma correlations between JOSs and restudy choices. Participants thus chose the pairs with poorer memory predictions more often for restudy. Surprisingly, however, this relationship was weaker in the delayed-JOS group than in the immediate-JOS group. This, too, suggests that restudy choices in the delayed-JOS group did not benefit that much from improved metamemory monitoring. Paradoxically, biased and erroneous metamemory monitoring in the immediate-JOS group affected restudy choices more strongly than debiased and more accurate metamemory monitoring in the delayed-JOS group did. These results indicate that the causal relationship between metamemory monitoring and control is not straightforward.

### Effects of Debiased Metamemory Control on Source Memory

Overall, metamemory monitoring and metamemory control of schema-based source memory were debiased and, thus, improved by delaying JOSs and eliciting source retrieval. Source memory was theorized to be affected by this debiased metamemory monitoring and control. As the delayed-JOS group restudied relatively more expected source–item pairs than the immediate-JOS group, we expected source memory for expected pairs to be better in the delayed-JOS group than in the immediate-JOS group. We predicted that this might attenuate or eliminate the inconsistency effect on source memory. Somewhat surprisingly, this was not the case.

Critically, delaying JOSs and restudy choices did not result in better source memory for expected source–item pairs (which is typically quite poor) compared with the immediate-JOS group. It did, however, result in worse source memory for unexpected pairs compared with the immediate-JOS group. This aligned with restudy choices: The delayed-JOS group restudied fewer unexpected pairs than the immediate-JOS group. Nonetheless, these restudy choices did not attenuate the inconsistency effect on source memory. One of our prior studies (Schaper & Bayen, 2021) suggested that biased restudy choices may contribute to the inconsistency effect. The current study does not confirm this.

One possible reason for the lack of source-memory differences between groups may be that remedying the expectancy illusion was relatively ineffective in the delayed-JOS group. The delayed-JOS group predicted a null effect of expectation on source memory, not an inconsistency effect. As delayed JOSs only partially remedied the expectancy illusion, the difference in restudy choices between the immediate and the delayed-JOS group may have been too weak to affect source memory. Completely remedying the expectancy illusion (i.e., participants predicting an inconsistency effect) might result in choosing more expected than unexpected pairs for restudy. A complete remedy might be accomplished, for example, by manipulating peoples’ beliefs prior to the experiment (e.g., Yan et al., 2016, for a remedy of the false conviction that blocked learning is more effective than interleaved learning) or by assigning point values to items in correspondence to their actual difficulty (e.g., Murphy et al., 2022, for a remedy of the font-size illusion). If participants restudied mostly expected pairs and very few (or no) unexpected pairs, source memory for expected pairs might benefit more strongly, and, thus, the inconsistency effect might be attenuated.

Rather than with biased restudy, the inconsistency effects in both groups may be explained by the attention-elaboration account (e.g., Erdfelder & Bredenkamp, 1998). Unexpected source–item pairs likely attracted more attention during study than expected source–item pairs and were, therefore, encoded more elaborately and remembered better. Such effects were not mitigated by differential restudy between the experimental groups. Therefore, restudy may be ineffective to improve schema-based source memory, or four seconds of restudy may be insufficient to affect memory substantially. Further, mere restudy of more expected pairs may not counteract the attention and elaboration benefits for unexpected source–item pairs as these same benefits may also occur at restudy (i.e., at the second encoding opportunity). Although participants selected these expected pairs for restudy, they may not have attended to them very well during restudy. Instructing participants to use control mechanisms other than restudying might thus more effectively improve source memory. For example, explicit instructions to attend to expected source–item pairs might enhance source memory for expected items.

This study may contribute to the understanding that the relationships between metamemory monitoring, metamemory control, and memory are not necessarily as straightforward as they have sometimes been portrayed (e.g., Dunlosky & Metcalfe, 2009; Dunlosky et al., 2005; Nelson et al., 2004; Thiede, 1999; Thiede et al., 2003). Regarding metamemory, we found that despite partially remedying the expectancy illusion in metamemory monitoring, metamemory control did not improve as much as it could have. We also found that the different restudy choices in the two groups only weakly affected source memory. Our study thus shows that improving metamemory monitoring is not always sufficient to also improve metamemory control and memory.

Other researchers have raised similar concerns. For example, Kimball et al., (2012) showed that delaying metamemory judgments and restudy choices did not improve cued recall. However, unlike our study, Kimball et al. did not correct metamemory illusions but investigated the general beneficial effect of delaying judgments on metamemory accuracy (see meta-analysis by Rhodes & Tauber 2011). Although correcting a strong metamemory illusion might be assumed to have a clearer effect on metamemory control and memory, this was not the case in our study. Further, Begg et al. (1992) showed that, whereas metamemory monitoring after a delay allowed participants to detect items that were not well learned, these items benefitted from restudy as much as items that participants deemed well learned. From our results, we may conclude that source memory does not benefit very much from the restudy of selected materials, even if the restudy choices are based on accurate metamemory monitoring. Thus, remedying metamemory illusions alone may not always be sufficient to improve learning behavior and memory. People may need to learn how to effectively translate their improved metamemory monitoring into metamemory control so that they select appropriate study strategies for the task at hand. Critically, they may further need to learn how to translate improved metamemory control into enhanced memory. In the future, other metamemory illusions should be studied to investigate the generalizability of our results.

## Appendix: Effects of Metamemory Monitoring and Control on Source Guessing

We want to comment on an interesting observation regarding source guessing not mentioned in the main text. When people do not remember the source of information in a source-monitoring task, they must guess the source (see Figure 4). Schema-based expectations typically bias source guessing toward the source that is schema-consistent with the test item. That is, if people do not remember the source of an item, they tend to guess that it originated from an expected source (Arnold et al., 2013; Bayen & Kuhlmann, 2011; Bayen et al., 2000; Bell et al., 2012; Ehrenberg & Klauer, 2005; Kroneisen & Bell, 2013; Kroneisen et al., 2015; Kuhlmann et al., 2012; Küppers & Bayen, 2014; Schaper & Bayen, 2021; Schaper et al., 2019a, b, 2021; Spaniol & Bayen, 2002; Wulff & Kuhlmann, 2020).

Therefore, we expected source guessing to be biased in favor of the expected source. To test this, we calculated the parameter difference Δ*g* = *g* – 0.50 (with *g* = .50 indicating unbiased source guessing and *g* >.50 indicating schema-consistent guessing). Participants guessed schema-consistently in the immediate-JOS group, Δ*g* = .25 [.16, .32], and in the delayed-JOS group, Δ*g* = .11 [< .01, .20]. Interestingly, guessing bias was significantly stronger in the immediate than in the delayed-JOS group, Δ*g*_immediate − delayed_ = .15 [.02, .28].

This group difference in source-guessing bias may be due to the difference in restudy choices. Group differences in restudy may have resulted in different metacognitive thoughts at test. The immediate-JOS group restudied more unexpected source–item pairs. At test, this may have resulted in the conviction that if an item had been presented with the unexpected source, the source would have been remembered. This may have strongly biased participants in the immediate-JOS group to guess the expected source when they did not remember the source. Conversely, in the delayed-JOS group, participants restudied about half expected and half unexpected source–item pairs. Thus, they likely lacked a similar conviction and their schema-consistent guessing bias was less pronounced.

## Footnotes

1 Note that the inconsistency effect on source memory mainly emerges when schema-based expectations are strongly violated. Mild violations of expectations have a comparably weak or no effect on source memory (Bayen & Kuhlmann, 2011; Bayen et al., 2000; Kuhlmann et al., 2012; Wulff & Kuhlmann, 2020).

2 In the source-monitoring paradigm, JOLs and JOSs are often similarly affected by experimental manipulations. However, there are also systematic differences between the two types of judgments. For example, the expectancy effect is consistently larger on JOSs than on JOLs (Mieth et al., 2021; Schaper et al., 2019a, b).

3 It should be noted that the schema-based source-monitoring paradigm shows similarities to a paired-associates paradigm using related and unrelated word pairs (such as dog – cat vs. dog – spoon, e.g., Undorf & Erdfelder, 2011). Both paradigms co-exist in the literature and are concerned with different aspects of episodic memory. The source-monitoring paradigm involves, among other processes, source memory for the item’s association with one of few possible sources. By contrast, the paired-associates paradigm typically involves cued recall, that is, a conglomerate of recalling the target and the cue-target association. The memory processes in the two paradigms are differently affected by expectancy or relatedness. For example, source memory is enhanced for unexpected pairs, whereas cued recall benefits from relatedness (e.g., Undorf & Erdfelder, 2011). However, metamemory is similarly affected by expectancy and relatedness: Metamemory judgments are typically higher for related words than unrelated words (e.g., Undorf & Erdfelder, 2011); and higher for expected sources than unexpected sources.

4 Under specific circumstances, people prioritize material for restudy that they deem better learned; for example when study time is severely limited (Metcalfe, 2002).

5 Participants could not restudy all pairs because this might have eliminated a possible difference between expected and unexpected pairs. Therefore, participants could select at most half of the pairs. Further, we wanted to avoid participants selecting no pairs for restudy. Therefore, they were told that they would restudy 32 pairs regardless of how many they chose (see Schaper & Bayen, 2021).

6 Only 71 participants’ data entered this analysis because one person in the immediate-JOS group made the same JOS for all items, leaving gamma undefined.

7 Only 71 participants’ data entered these analyses because one participant never incorrectly attributed an item to the unexpected source and therefore had zero observations in this response category.

## References

[CR1] Alba JW, Hasher L (1983). Is memory schematic?. Psychological Bulletin.

[CR2] Arnold NR, Bayen UJ, Kuhlmann BG, Vaterrodt B (2013). Hierarchical modeling of contingency-based source monitoring: A test of the probability-matching account. Psychonomic Bulletin & Review.

[CR3] Batchelder WH, Riefer DM (1990). Multinomial processing models of source monitoring. Psychological Review.

[CR4] Bayen UJ, Kuhlmann BG (2011). Influences of source–item contingency and schematic knowledge on source monitoring: Tests of the probability-matching account. Journal of Memory and Language.

[CR5] Bayen UJ, Murnane K, Erdfelder E (1996). Source discrimination, item detection, and multinomial models of source monitoring. Journal of Experimental Psychology: Learning Memory and Cognition.

[CR6] Bayen UJ, Nakamura GV, Dupuis SE, Yang CL (2000). The use of schematic knowledge about sources in source monitoring. Memory & Cognition.

[CR7] Begg IM, Anas A, Farinacci S (1992). Dissociation of processes in belief: Source recollection, statement familiarity, and the illusion of truth. Journal of Experimental Psychology: General.

[CR8] Bell R, Buchner A, Kroneisen M, Giang T (2012). On the flexibility of social source memory: A test of the emotional incongruity hypothesis. Journal of Experimental Psychology: Learning Memory and Cognition.

[CR9] Bell R, Mieth L, Buchner A (2015). Appearance-based first impressions and person memory. Journal of Experimental Psychology: Learning Memory and Cognition.

[CR10] Brewer WF, Treyens JC (1981). Role of schemata in memory for places. Cognitive Psychology.

[CR11] Bröder A, Meiser T (2007). Measuring source memory. Journal of Psychology.

[CR12] Bui Y, Pyc MA, Bailey H (2018). When people’s judgments of learning (JOLs) are extremely accurate at predicting subsequent recall: the “Displaced-JOL effect". Memory (Hove, England).

[CR13] Dodson CS, Darragh J, Williams A (2008). Stereotypes and retrieval-provoked illusory source recollections. Journal of Experimental Psychology: Learning Memory and Cognition.

[CR14] Dunlosky J, Hertzog C, Kennedy MRF, Thiede KW (2005). The self-monitoring approach for effective learning. Cognitive Technology.

[CR15] Dunlosky, J., & Metcalfe, J. (2009). *Metacognition*. Sage Publications, Inc.

[CR16] Dunlosky J, Nelson TO (1992). Importance of the kind of cue for judgments of learning (JOL) and the delayed-JOL effect. Memory & Cognition.

[CR17] Dunlosky J, Nelson TO (1994). Does the sensitivity of judgements of learning (JOLs) to the effects of various study activities depend on when the JOLs occur?. Journal of Memory and Language.

[CR18] Dunlosky J, Nelson TO (1997). Similarity between the cue for judgments of learning (JOL) and the cue for test is not the primary determinant of JOL accuracy. Journal of Memory and Language.

[CR19] Ehrenberg K, Klauer KC (2005). Flexible use of source information: Processing components of the inconsistency effect in person memory. Journal of Experimental Social Psychology.

[CR20] Erdfelder E, Bredenkamp J (1998). Recognition of script-typical versus script-atypical information: Effects of cognitive elaboration. Memory & Cognition.

[CR21] Finley JR, Tullis JG, Benjamin AS, Knine MS, Saleh IM (2010). Metacognitive control of learning and remembering. New Science of Learning.

[CR22] Friedman A (1979). Framing pictures: The role of knowledge in automatized encoding and memory for gist. Journal of experimental psychology: General.

[CR23] Gelman A, Rubin DB (1992). Inference from iterative simulation using multiple sequences. Statistical Science.

[CR24] Heck DW, Arnold NR, Arnold D (2018). TreeBUGS: An R package for hierarchical multinomial-processing-tree modeling. Behavior Research Methods.

[CR25] Hicks JL, Cockman DW (2003). The effect of general knowledge on source memory and decision processes. Journal of Memory and Language.

[CR26] Johnson MK, Hashtroudi S, Lindsay DS (1993). Source monitoring. Psychological Bulletin.

[CR27] Kelemen WL (2000). Metamemory cues and monitoring accuracy: Judging what you know and what you will know. Journal of Educational Psychology.

[CR28] Kelemen WL, Weaver CA (1997). Enhanced memory at delays: Why do judgments of learning improve over time?. Journal of Experimental Psychology: Learning Memory and Cognition.

[CR29] Kimball DR, Metcalfe J (2003). Delaying judgments of learning affects memory, not metamemory. Memory & Cognition.

[CR30] Kimball DR, Smith TA, Muntean WJ (2012). Does delaying judgments of learning really improve the efficacy of study decisions? Not so much. Journal of Experimental Psychology: Learning Memory and Cognition.

[CR31] Klauer KC (2010). Hierarchical multinomial processing tree models: A latent-trait approach. Psychometrika.

[CR32] Konopka AE, Benjamin AS (2009). Schematic knowledge changes what judgments of learning predict in a source memory task. Memory & Cognition.

[CR33] Koriat A (1997). Monitoring one’s own knowledge during study: A cue-utilization approach to judgments of learning. Journal of Experimental Psychology: General.

[CR34] Koriat A, Bjork RA (2006). Illusions of competence during study can be remedied by manipulations that enhance learners’ sensitivity to retrieval conditions at test. Memory & Cognition.

[CR35] Koriat A, Bjork RA (2006). Mending metacognitive illusions: A comparison of mnemonic-based and theory-based procedures. Journal of Experimental Psychology: Learning Memory and Cognition.

[CR36] Koriat A, Ma’ayan H (2005). The effects of encoding fluency and retrieval fluency on judgments of learning. Journal of memory and Language.

[CR37] Kornell, N., & Finn, B. (2016). Self-regulated learning: An overview of theory and data. In J. Dunlosky & S. K. Tauber (Eds.), *The Oxford handbook of metamemory* (pp. 325-354). Oxford University Press. 10.1093/oxfordhb/9780199336746.013.23

[CR38] Kranz D, Nadarevic L, Erdfelder E (2019). Bald and bad? Experimental evidence for a dual-process account of baldness stereotyping. Experimental Psychology.

[CR39] Kroneisen M, Bell R (2013). Sex, cheating, and disgust: Enhanced source memory for trait information that violates gender stereotypes. Memory (Hove, England).

[CR40] Kroneisen M, Woehe L, Rausch LS (2015). Expectancy effects in source memory: How moving to a bad neighborhood can change your memory. Psychonomic Bulletin & Review.

[CR41] Kuhlmann BG, Bayen UJ, Dunlosky J, Tauber SK (2016). Metacognitive aspects of source monitoring. The Oxford Handbook of Metamemory.

[CR42] Kuhlmann BG, Vaterrodt B, Bayen UJ (2012). Schema bias in source monitoring varies with encoding conditions: Support for a probability-matching account. Journal of Experimental Psychology: Learning Memory and Cognition.

[CR43] Küppers V, Bayen UJ (2014). Inconsistency effects in source memory and compensatory schema-consistent guessing. The Quarterly Journal of Experimental Psychology.

[CR44] Loftus GR, Mackworth NH (1978). Cognitive determinants of fixation location during picture viewing. Journal of Experimental Psychology: Human Perception and Performance.

[CR45] Loftus GR, Masson MEJ (1994). Using confidence intervals in within-subject designs. Psychonomic Bulletin & Review.

[CR46] Luna K, Martín-Luengo B, Albuquerque PB (2018). Do delayed judgements of learning reduce metamemory illusions? A meta-analysis. Quarterly Journal of Experimental Psychology.

[CR47] Marsh R, Cook G, Hicks JL (2006). Gender and orientation stereotypes bias source-monitoring attributions. Memory (Hove, England).

[CR48] Mather M, Johnson MK, De Leonardis DM (1999). Stereotype reliance in source monitoring: Age differences and neuropsychological test correlates. Cognitive Neuropsychology.

[CR49] Meeter M, Nelson TO (2003). Multiple study trials and judgments of learning. Acta Psychologica.

[CR50] Metcalfe J (2002). Is study time allocated selectively to a region of proximal learning?. Journal of Experimental Psychology: General.

[CR51] Metcalfe J, Finn B (2008). Evidence that judgments of learning are causally related to study choice. Psychonomic Bulletin & Review.

[CR52] Mieth L, Bell R, Buchner A (2016). Facial likability and smiling enhance cooperation, but have no direct effect on moralistic punishment. Experimental Psychology.

[CR53] Mieth L, Schaper ML, Kuhlmann BG, Bell R (2021). Memory and metamemory for social interactions: Evidence for a metamemory expectancy illusion. Memory & Cognition.

[CR54] Murnane K, Bayen UJ (1996). An evaluation of empirical measures of source identification. Memory and Cognition.

[CR55] Murnane K, Bayen UJ (1998). Measuring memory for source: Some theoretical assumptions and technical limitations. Memory & Cognition.

[CR56] Murphy DH, Huckins SC, Rhodes MG, Castel AD (2022). The effect of perceptual processing fluency and value on metacognition and remembering. Psychonomic Bulletin & Review.

[CR57] Nelson TO (1984). A comparison of current measures of the accuracy of feeling-of-knowing predictions. Psychological bulletin.

[CR58] Nelson TO, Dunlosky J (1991). When people’s judgments of learning (JOLs) are extremely accurate at predicting subsequent recall: The “delayed-JOL effect". Psychological Science.

[CR59] Nelson TO, Dunlosky J, Graf A, Narens L (1994). Utilization of metacognitive judgments in the allocation of study during multitrial learning. Psychological Science.

[CR60] Nelson, T. O., & Narens, L. (1990). Metamemory: A theoretical framework and new findings. In G. H. Bower (Ed.), *The psychology of learning and motivation* (Vol. 26, pp. 125–141). Academic Press. 10.1016/S0079-7421(08)60053-5

[CR61] Nelson TO, Narens L, Dunlosky J (2004). A revised methodology for research on metamemory: Pre-judgment recall and monitoring (PRAM). Psychological methods.

[CR62] Plummer, M. (2003). JAGS: A program for analysis of Bayesian graphical models using Gibbs sampling. In In K. Hornik, F. Leisch, & A. Zeileis (Eds.),* Proceedings of the 3rd international workshop on distributed statistical computing* (Vol. 124, pp. 1–10). Technische Universität Wien. http://www.ci.tuwien.ac.at/Conferences/DSC-2003/Drafts/Plummer.pdf

[CR63] Pyc MA, Rawson KA, Aschenbrenner AJ (2014). Metacognitive monitoring during criterion learning: When and why are judgments accurate?. Memory & Cognition.

[CR64] Rhodes MG, Dunlosky J, Tauber SU (2016). Judgments of learning: Methods, data, and theory. The Oxford Handbook of Metamemory.

[CR65] Rhodes MG, Castel AD (2008). Memory predictions are influenced by perceptual information: Evidence for metacognitive illusions. Journal of Experimental Psychology: General.

[CR66] Rhodes MG, Tauber SK (2011). The influence of delaying judgments of learning on metacognitive accuracy: A meta-analytic review. Psychological Bulletin.

[CR67] Schaper ML, Bayen UJ (2021). The metamemory expectancy illusion in source monitoring affects metamemory control and memory. Cognition.

[CR68] Schaper, M. L., Bayen, U. J., & Hey, C. V. (2021). Delaying metamemory judgments corrects the expectancy illusion in source monitoring: The role of fluency and belief. *Journal of Experimental Psychology: Learning, Memory, and Cognition.* Advance online publication. 10.1037/xlm0001088 10.1037/xlm0001088 34726439

[CR69] Schaper ML, Kuhlmann BG, Bayen UJ (2019). Metamemory expectancy illusion and schema-consistent guessing in source monitoring. Journal of Experimental Psychology: Learning Memory and Cognition.

[CR70] Schaper ML, Kuhlmann BG, Bayen UJ (2019). Metacognitive expectancy effects in source monitoring: Beliefs, in-the-moment experiences, or both?. Journal of Memory and Language.

[CR71] Sherman JW, Bessenoff GR (1999). Stereotypes as source-monitoring cues: On the interaction between episodic and semantic memory. Psychological Science.

[CR72] Sherman, J. W., Lee, A. Y., Bessenoff, G. R., & Frost, L. A. (1998). Stereotype efficiency reconsidered: Encoding flexibility under cognitive load. *Journal of Personality and Social Psychology*, *75*(3), 589–606. 10.1037/0022-3514.75.3.589 10.1037//0022-3514.75.3.589 9781404

[CR73] Shi LZ, Tang WH, Liu XP (2012). Age-related schema reliance of judgments of learning in predicting source memory. Aging Neuropsychology and Cognition.

[CR74] Spaniol J, Bayen UJ (2002). When is schematic knowledge used in source monitoring?. Journal of Experimental Psychology: Learning Memory and Cognition.

[CR75] Thiede KW (1999). The importance of monitoring and self-regulation during multitrial learning. Psychonomic Bulletin & Review.

[CR76] Thiede KW, Anderson M, Therriault D (2003). Accuracy of metacognitive monitoring affects learning of texts. Journal of Educational Psychology.

[CR77] Tullis JG, Finley JR, Benjamin AS (2013). Metacognition of the testing effect: Guiding learners to predict the benefits of retrieval. Memory & Cognition.

[CR78] Undorf, M. (2020). Fluency illusions in metamemory. In A.M. Cleary & B.L. Schwartz (Eds.), *Memory quirks: The study of odd phenomena in memory* (1st ed., pp. 150–174). Routledge. 10.4324/9780429264498

[CR79] Undorf M, Erdfelder E (2011). Judgments of learning reflect encoding fluency: Conclusive evidence for the ease-of-processing hypothesis. Journal of Experimental Psychology: Learning Memory and Cognition.

[CR80] Van Overschelde JP, Nelson TO (2006). Delayed judgments of learning cause both a decrease in absolute accuracy (calibration) and an increase in relative accuracy (resolution). Memory & cognition.

[CR81] Weaver CA, Kelemen WL (1997). Judgments of learning at delays: Shifts in response patterns or increased metamemory accuracy?. Psychological Science.

[CR82] Wulff L, Kuhlmann BG (2020). Is knowledge reliance in source guessing a cognitive trait? Examining stability across time and domain. Memory & Cognition.

[CR83] Yan VX, Bjork EL, Bjork RA (2016). On the difficulty of mending metacognitive illusions: A priori theories, fluency effects, and misattributions of the interleaving benefit. Journal of Experimental Psychology: General.

[CR84] Yang C, Potts R, Shanks DR (2017). Metacognitive unawareness of the errorful generation benefit and its effects on self-regulated learning. Journal of Experimental Psychology: Learning Memory and Cognition.

[CR85] Dunlosky John, Mueller Michael L., Morehead Kayla, Tauber Sarah K., Thiede Keith W., Metcalfe Janet (2021). Why Does Excellent Monitoring Accuracy Not Always Produce Gains in Memory Performance?. Zeitschrift für Psychologie.

